# Correlational patterns of neuronal activation and epigenetic marks in the basolateral amygdala and piriform cortex following olfactory threat conditioning and extinction in rats

**DOI:** 10.3389/fnmol.2024.1355140

**Published:** 2024-03-14

**Authors:** Tian Qin, Yue Xia, Negar Nazari, Tayebeh Sepahvand, Qi Yuan

**Affiliations:** Division of Biomedical Sciences, Faculty of Medicine, Memorial University, St. John’s, NL, Canada

**Keywords:** olfactory, threat conditioning, extinction, amygdala, piriform cortex, DNA methylation, histone acetylation, cFos

## Abstract

**Introduction:**

Cumulative evidence suggests that sensory cortices interact with the basolateral amygdala (BLA) defense circuitry to mediate threat conditioning, memory retrieval, and extinction learning. The olfactory piriform cortex (PC) has been posited as a critical site for olfactory associative memory. Recently, we have shown that N-methyl-D-aspartate receptor (NMDAR)-dependent plasticity in the PC critically underpins olfactory threat extinction. Aging-associated impairment of olfactory threat extinction is related to the hypofunction of NMDARs in the PC.

**Methods:**

In this study, we investigated activation of neuronal cFos and epigenetic marks in the BLA and PC using immunohistochemistry, following olfactory threat conditioning and extinction learning in rats.

**Results:**

We found highly correlated cFos activation between the posterior PC (pPC) and BLA. cFos was correlated with the degree of behavioral freezing in the pPC in both adult and aged rats, and in the BLA only in adult rats. Markers of DNA methylation 5 mC and histone acetylation H3K9/K14ac, H3K27ac, and H4ac exhibited distinct training-, region-, and age-dependent patterns of activation. Strong correlations of epigenetic marks between the BLA and pPC in adult rats were found to be a general feature. Conversely, aged rats only exhibited correlations of H3 acetylations between the two structures. Histone acetylation varied as a function of aging, revealed by a reduction of H3K9/K14ac and an increase of H4ac in aged brains at basal condition and following threat conditioning.

**Discussion:**

These findings underscore the coordinated role of PC and BLA in olfactory associative memory storage and extinction, with implications for understanding aging related cognitive decline.

## 1 Introduction

The threat conditioning and extinction paradigm in rodents has been a successful model for maladaptive fear learning and associated exposure therapy in humans. The basolateral amygdala (BLA) serves as a core component for contextual and cue-associated threat conditioning and extinction (Fanselow and LeDoux, 1999; Bouton et al., 2021; Sepahvand et al., 2023b). This structure interacts with the prefrontal cortex, hippocampus, sensory and neuromodulatory structures to form a larger network of threat circuitry (Bouton et al., 2021; Sepahvand et al., 2023b). As a central hub for threat memory encoding, the BLA converges sensory inputs with aversive stimuli to form long-term potentiation (LTP) at the sensory input synapses, leading to heightened output from the central amygdala defense pathways upon memory recall (Sigurdsson et al., 2007; Pape and Pare, 2010; Johansen et al., 2011; Sepahvand et al., 2023b). Extinction is acquired through repeat exposure to a non-reinforced sensory conditioned stimulus following threat conditioning. Extinction training engages either depotentiation of the synaptic LTP in the BLA, or new inhibitory learning processes to suppress the original threat memory (Bouton et al., 2021; Sepahvand et al., 2023b). The latter is achieved through a coordination of circuitry which includes inhibitory neurons of the amygdala, hippocampus and infralimbic prefrontal cortex (Corcoran and Maren, 2001; Herry and Mons, 2004; Burgos-Robles et al., 2007; Mueller et al., 2008; Sotres-Bayon et al., 2009; Lacagnina et al., 2019). Noradrenergic and dopaminergic inputs to the BLA facilitate threat encoding (Guarraci et al., 1999; Bouret et al., 2003; Johansen et al., 2014; Soya et al., 2017) and engage in prediction-error signaling in extinction learning (Mueller et al., 2008; Luo et al., 2018; Salinas-Hernandez et al., 2018)

Emergent research suggests that sensory cortices are critically involved in threat conditioning (Sacco and Sacchetti, 2010; Hegoburu et al., 2014; Cambiaghi et al., 2016b; Meissner-Bernard et al., 2019; East et al., 2021; Mouly et al., 2022; You et al., 2022) and extinction of threat memory (Sepahvand et al., 2023a). Particularly, the olfactory piriform cortex (PC) has extensive mutual connections with the BLA, and has been suggested to be a more permanent repertoire for olfactory threat memory storage (Li and Wilson, 2023; Sepahvand et al., 2023b). Synchronized neuronal activity in the PC and BLA underlies olfactory threat memory formation and ensures precision of encoding (East et al., 2021). Recently, we have reported that the posterior PC (pPC) critically mediates olfactory threat extinction through NMDAR-dependent long-term depression (LTD) (Sepahvand et al., 2023a). Aging-associated impairment in olfactory threat extinction is related to the hypofunction of NMDARs in the PC (Sepahvand et al., 2023a). However, it is not known whether and how the BLA and PC coordinate in olfactory extinction learning, and how their coordination is affected by aging.

Chromatin modifications such as DNA methylation and histone acetylation regulate gene repression and activation in the brain, which critically underpin adaptive behavior (Graff and Tsai, 2013; Guzman-Karlsson et al., 2014). Epigenetic mechanisms have been implicated in threat conditioning in the BLA. For instance, auditory threat conditioning and extinction are associated with increased histone acetylation in this region, which is further enhanced by inhibitors of histone deacetylase or DNA methyltransferase (Monsey et al., 2011; Itzhak et al., 2012; Siddiqui et al., 2017). Thus, epigenetic mechanisms play a pivotal role in governing synaptic plasticity, as well as the consolidation and extinction processes of threat memory within the BLA. Epigenetic changes in olfactory threat learning and extinction have not been characterized. In this study, we investigated whether BLA and PC synchronize their neuronal activity during olfactory threat memory and extinction, using cFos as a marker. Additionally, we explored whether these two structures demonstrate comparable patterns of epigenetic alterations in adult and aged rats. Our results elucidate the intricate interplay between the PC and BLA in mediating olfactory threat memory and extinction, and highlight age-related alterations in epigenetic regulation.

## 2 Material and methods

### 2.1 Animal behavioral experiments

#### 2.1.1 Subjects

**FIGURE 1:**
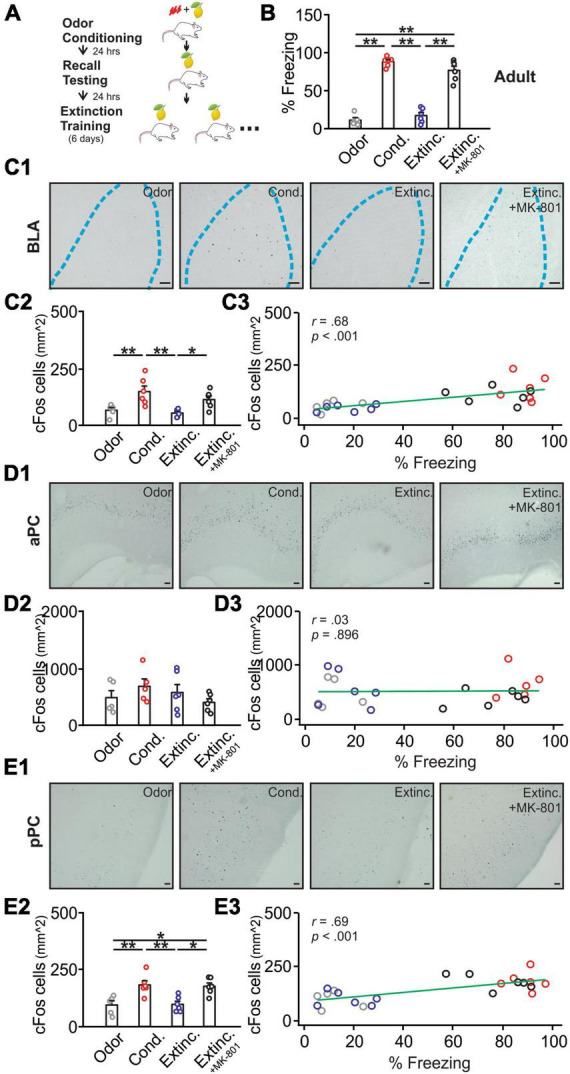
cFos activations in the basolateral amygdala (BLA), posterior piriform cortex (pPC), but not anterior piriform cortex (aPC), were strongly correlated with behavioral freezing scores in adult rats. **(A)** Schematics of behavioral procedures. **(B)** The percentage of freezing to the conditioned odor in 5 min in four groups: odor only (Odor), odor conditioned (Cond.), extinction-trained (Extinc.) and extinction-trained plus MK-801 injections (Extinc. + MK-801). **(C1)** Example images of cFos expression in the BLA. **(C2)** cFos^+^ cell counts in the BLA. **(C3)** The correlation between cFos^+^ cell counts in the BLA and percentage freezing. **(D1)** Example images of cFos expression in the aPC. **(D2)** cFos^+^ cell counts in the aPC. **(D3)** The correlation between cFos^+^ cell counts in the aPC and percentage freezing. **(E1)** Example images of cFos expression in the pPC. **(E2)** cFos^+^ cell counts in the pPC. **(E3)** The correlation between cFos^+^ cell counts in the pPC and percentage freezing. Scale bars, 50 μm. *p < 0.05; **p < 0.01.

**FIGURE 2:**
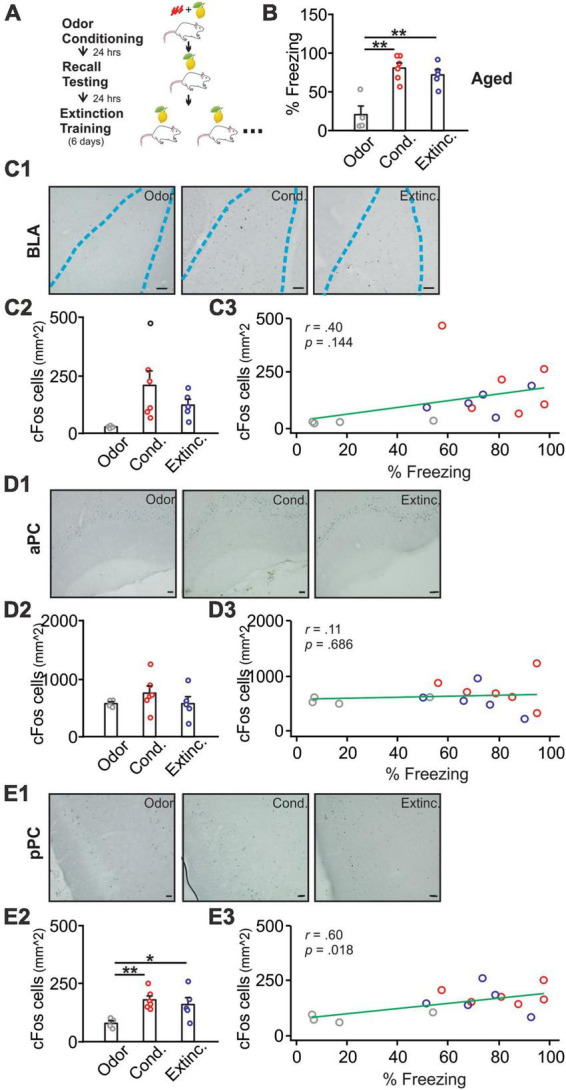
cFos activations in the BLA, pPC, but not aPC, were strongly correlated with behavioral freezing scores in aged rats. **(A)** Schematics of behavioral procedures. **(B)** The percentage of freezing to the conditioned odor in 5 min in three groups: odor only (Odor), odor conditioned (Cond.), and extinction-trained (Extinc.). **(C1)** Example images of cFos expression in the BLA. **(C2)** cFos^+^ cell counts in the BLA. **(C3)** The correlation between cFos^+^ cell counts in the BLA and percentage freezing. **(D1)** Example images of cFos expression in the aPC. **(D2)** cFos^+^ cell counts in the aPC. **(D3)** The correlation between cFos^+^ cell counts in the aPC and percentage freezing. **(E1)** Example images of cFos expression in the pPC. **(E2)** cFos^+^ cell counts in the pPC. **(E3)** The correlation between cFos^+^ cell counts in the pPC and percentage freezing. Scale bars, 50 μm. *p < 0.05; **p < 0.01.

A total of 23 adult rats (3–6 months-old) and 15 aged Sprague-Dawley rats (18–24 months-old) of both sexes were used in the behavioral experiments. Our previous research reported no sex difference in the olfactory threat learning and extinction at both ages (Sepahvand et al., 2023a). Partial behavioral data (Figures 1B, 2B), associated PC cFos analysis (Figures 1D–E, 2D–E) and brain tissue were obtained through a previous project (Sepahvand et al., 2023a). Rats were accommodated in a controlled environment featuring a standard 12-h light-dark cycle, with *ad libitum* access to food and water. All behavioral manipulations were completed during the light phase of the light cycle. All experimental protocols were approved by the Institutional Animal Care Committee at Memorial University of Newfoundland and adhered to the guidelines for Animal Care set forth by the Canadian Council.

Four groups of adult rats were included: (a) an odor-only group (*N* = 5; 3M/2F) in which rats were exposed to only odor without shock during training; (b) a conditioned group (*N* = 6; 4M/2F) in which rats underwent paired odor and shock training; (c) an extinction-trained group (*N* = 6; 3M/3F) in which rats underwent 6 days of extinction training, following the odor and shock conditioning; and (d) an extinction-trained group which received an injection of the NMDAR antagonist MK-801 (*N* = 6, 2M/4F). For the aged rats, three groups were included: (a) an odor-only group (*N* = 4M); (b) a conditioned group (*N* = 6, 5M/1F); and (c) an extinction-trained group (*N* = 5; 4M/1F). Immunohistochemistry (IHC) analysis of cFos and various epigenetic markers were conducted using the brain tissue from the same rats that underwent behavioral training and testing.

#### 2.1.2 Odorants

Odorant terpenes (6.63%, 1 Pascal of vapor pressure) implemented in the behavioral experiment was diluted with mineral oil, which was chosen for its demonstrated affective neutrality in adult rats (Devore et al., 2013).

#### 2.1.3 Apparatus

All phases of the behavioral training and testing took place within a shock box which was attached to a custom-designed olfactometer that allowed for controlled delivery of both air and the odorant. The shock box was composed of a Plexiglas chamber with an electrified grid underneath, connected to the shock generator (Muromachi Kikai Model SGS-003DX, Japan or San Diego Instruments, United States). The odorant was stored in a polyvinyl carbonate bottle and connected to the olfactometer via C-flex tubing, which was securely clamped when not in use. An evacuation fan system was connected to the top lid of the shock chamber to expedite odor clearance and prevent odor contamination.

#### 2.1.4 Olfactory threat conditioning

Rats were habituated in the shock box for two consecutive days. On the third day, rats were individually trained. Individual training sessions consisted of four distinct exposures. Each exposure involved either the paired presentation of the odor with a foot shock, or the presentation of the odor with an absence of a foot shock. The odor stimulus was administered at the 5-, 15-, 20-, and 30-min marks within the 30-min training session, with each odor presentation lasting for one minute. The foot shock coincided with the final second of each 60 s odor delivery (0.5 mA for 1 s) in the conditioning group. The fourth day was recall day, wherein rats were introduced to the shock chamber without any administration of shock or odor stimulus for 5 min. This was followed by exposure to the odor stimulus for 5 min. The memory test sessions were videotaped, and freezing scores were analyzed offline. Freezing was operationally defined as the absence of any movement except respiration-related motions. The percentage of freezing was calculated during the baseline and five-minute odor exposure. Experiments involving both sexes were conducted in an intermixed fashion, with the order of male and female rats randomized. Between each trial of the experiment, the shock chamber and grids were thoroughly cleaned with 70% ethanol and clean paper towels.

#### 2.1.5 Olfactory threat extinction

Twenty-four hours following the recall of odor threat memory, the adult and aged rat extinction groups underwent six consecutive days of extinction training. Each extinction session consisted of spending the first 5 min in the shock box without odor presentation, while during the last 5 min, the odor was continuously presented. Experiments were videotaped and freezing was counted offline blindly. The percentage of freezing was calculated during the baseline and 5-minute odor exposure.

#### 2.1.6 Drug administration

NMDAR antagonist MK-801 (0.05 mg/kg, Tocris) solution was freshly prepared on testing days by dissolution in sterile 0.9% saline (Sepahvand et al., 2023a). The drug was subcutaneously administered to the rats immediately after the recall test of odor threat conditioning, and 30 min prior to each extinction session to test the effect of NMDAR blockade on threat extinction.

### 2.2 IHC and imaging analysis

Rats were transcardially perfused 90 min post behavioral testing, with ice-cold saline followed by 4% paraformaldehyde in PBS and brains were extracted. Brains were sectioned into 50 μm-thick coronal slices using a vibratome (Leica VT 1000P; Leica Biosystems) or compresstome (Precision Instruments). Slices were stored in PVP solution at −4°C until further processing. Sections from the BLA, anterior PC (aPC) and pPC with similar anterior-posterior (AP) coordinates were selected for IHC. cFos and epigenetic marker IHC were conducted using the same animals, following behavioral tests.

#### 2.2.1 Antibodies

c-Fos antibody (Rabbit mAb #2250, Cell Signaling) was used to assess neuronal activation. 5-Methylcytosine antibody (Rabbit mAb #28692, Cell Signaling) was used to evaluate methylation levels on the DNA base cytosine. H3K9/K14Ac antibody (Rabbit #9677, Cell Signaling) was used to evaluate the acetylation at the Lysine 9 and Lysine 14 sites of the H3 histone. H3K27Ac antibody (Rabbit mAb #16602) was used to evaluate the acetylation modifications on the lysine 27 site of H3 histone. Pan-H4Ac antibody (Rabbit #39925, Active Motif) was used to evaluate various lysine acetylation positions in the H4 histone.

#### 2.2.2 IHC protocol

The sections were washed 10 × 2 min in Tris buffer (0.1M, pH 7.6), followed by a 30 min incubation in 0.03% H_2_O_2_ in Tris buffer. This was followed by a 10 min exposure to Tris A (0.1% TritonX in Tris buffer) and Tris B (0.1% TritonX and 0.005% BSA in Tris buffer), prior to the application of a blocking solution consisting of 10% normal goat serum (Sigma-Aldrich) for a duration of 1 h. Subsequently, the sections underwent a ten-minute wash in each of Tris A and Tris B before incubating in the primary antibodies (1:10,000) solution prepared in Tris B overnight. Following the ten minute washes in Tris A and Tris B, the sections were treated with a goat anti-rabbit biotinylated secondary antibody solution (1:1,000, ThermoFisher), which had been prepared in Tris B and kept at 4°C for 45 min. After further washing in Tris B and Tris D (0.1 Triton X and 0.005% BSA in 0.5 M Tris buffer), the sections were immersed in avidin–biotin horseradish peroxidize complex (1:1000 in Tris D; Vector ABC kit, Vector Laboratories) for 1.5 h at room temperature, followed by three successive rinses with Tris buffer. Sections were then incubated with peroxidase substrate (Vector SG) for 5 min. Nuclei displaying cFos or epigenetic marker positivity exhibited a blue-gray staining. Sections were first washed in Tris buffer for 5 min, then mounted on slides, air-dried, dehydrated in ethanol solutions and xylene, and coverslipped with Permount (Fisher).

#### 2.2.3 Imaging and data analysis

Images were acquired by bright-field microscopy (10x, BX53 Upright Microscope, Olympus) using CellSens standard software. Uniform standardization of light intensity and exposure parameters was applied to all captured images. ImageJ software was used for cell counting and relative optical density (ROD) measurement.

Three-to-six sections of BLA, aPC and pPC (layer II/III) regions with comparable AP coordinates from each animal were used for analysis, and data were averaged for each subject. For cFos, ImageJ software was employed to count the cfos-positive nuclei. Images underwent background subtraction before automatic cell counting using the Trainable Weka Segmentation plugin. The quantity of c-Fos immunoreactive neurons were then normalized to the region of interest (ROI, mm^2^). For epigenetic markers, the mean gray density of the ROI was measured and normalized to the background density value.

### 2.3 Statistics

OriginPro 2022b software was utilized for data analysis. Multiple-group comparisons were conducted through one-way ANOVAs, followed by *post hoc* Fisher tests. The Pearson correlation coefficient was used to assess any correlation between cFos and freezing scores, cFos and epigenetic markers in each structure, and epigenetic markers between the BLA and PC. Statistical significance was determined at a p-value of less than alpha set at 0.05. The results were presented as means ± SEM.

## 3 Results

### 3.1 Neuronal activation in the BLA, pPC, but not aPC were correlated with freezing scores

Figure 1A illustrates the training schematics. Adult rats [*F*(3,19) = 96.38, *p* < 0.001] showed a high percentage of freezing following olfactory threat conditioning and reduced freezing to the control level following extinction training. The extinction group with MK-801 injection, however, failed to extinguish the olfactory threat memory (Figure 1B). The freezing to the odor was significantly higher than the baseline freezing in the conditioned group, suggesting that the conditioned odor, rather than the context, induced-freezing dominated (Supplementary Figure 1). cFos expression in the BLA [*F*(3,19) = 7.15, *p* = 0.002; Figures 1C1, C2] followed the behavioral patterns. BLA cFos^+^ nuclei were significantly higher in the conditioned group and reduced to control levels following extinction training. The MK-801-injected group maintained a high level of cFos expression, comparable to the conditioned group. There was a significant correlation between cFos expression in the BLA and percentage of freezing in rats (*r* = 0.68, *p* < 0.001; Figure 1C3). Conversely, cFos expression in the aPC did not differ among groups (*F*(3,18) = 1.07, *p* = 0.385; Figures 1D1, D2), and was not correlated with freezing behavior (*r* = 0.03, *p* = 0.896; Figure 1D3). However, cFos patterns in the pPC, showed similar patterns to those of the BLA [*F*(3,19) = 8.82, *p* < 0.001; Figures 1E1, E2], and correlated significantly with freezing scores of the rats (*r* = 0.69, *p* < 0.001; Figure 1E3).

Aged rats [*F*(2,14) = 15.46, *p* < 0.001] showed successful olfactory threat conditioning but impaired extinction learning (Figures 2A, B). Previous research suggests that impaired olfactory threat learning in aged rats is due to the lack of NMDAR-dependent LTD in the PC (Sepahvand et al., 2023a). cFos expression in the BLA showed a trend. However, the differences among groups were not significant [*F*(2,12) = 3.53, *p* = 0.062; Figures 2C1, C2], and correlation with freezing scores was weak (*r* = 0.40, *p* = 0.144; Figure 2C3). cFos expression in the aPC showed no differences among groups [*F*(2,12) = 0.93, *p* = 0.422; Figures 2D1, D2], and was not correlated with freezing scores (*r* = 0.11, *p* = 0.686; Figure 2D3). In contrast, cFos patterns in the pPC paralleled the behavioral freezing [*F*(2,12) = 5.64, *p* = 0.019; Figures 2E1, E2], with higher levels observed in the conditioned and extinction-trained groups. cFos expression in the pPC was significantly correlated with the freezing scores (*r* = 0.60, *p* = 0.018; Figure 2E3).

**FIGURE 3:**
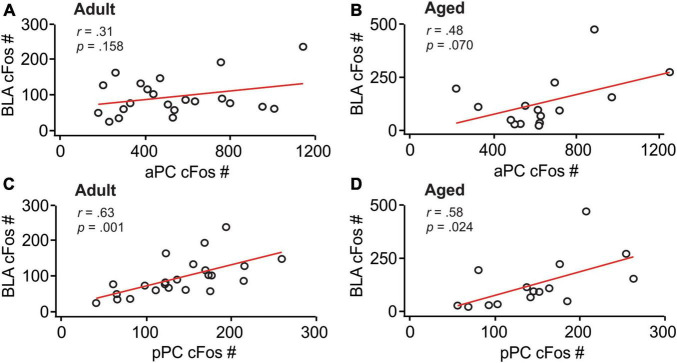
BLA and pPC cFos activations were significantly correlated. **(A)** The correlation between the aPC and BLA in adult rats. **(B)** The correlation between the aPC and BLA in aged rats. **(C)** The correlation between the pPC and BLA in adult rats. **(D)** The correlation between the pPC and BLA in aged rats.

We then analyzed the correlations of cFos patterns between these structures. aPC cFos^+^ nuclei numbers correlated with neither BLA cfos^+^ number in adult rats (*r* = 0.31, *p* = 0.158; Figure 3A), nor in aged rats (*r* = 0.48, *p* = 0.070; Figure 3B). In contrast, pPC cFos^+^ nuclei numbers were significantly correlated with BLA cFos^+^ numbers in both adult (*r* = 0.63, *p* = 0.001; Figure 3C) and aged (*r* = 0.58, *p* = 0.024; Figure 3D) rats. Together, these results suggest that neuronal activity in the BLA and pPC are synchronized to support olfactory threat memory encoding and extinction learning.

### 3.2 DNA methylation 5mC expression were correlated between the BLA and pPC in adult rats only

**FIGURE 4:**
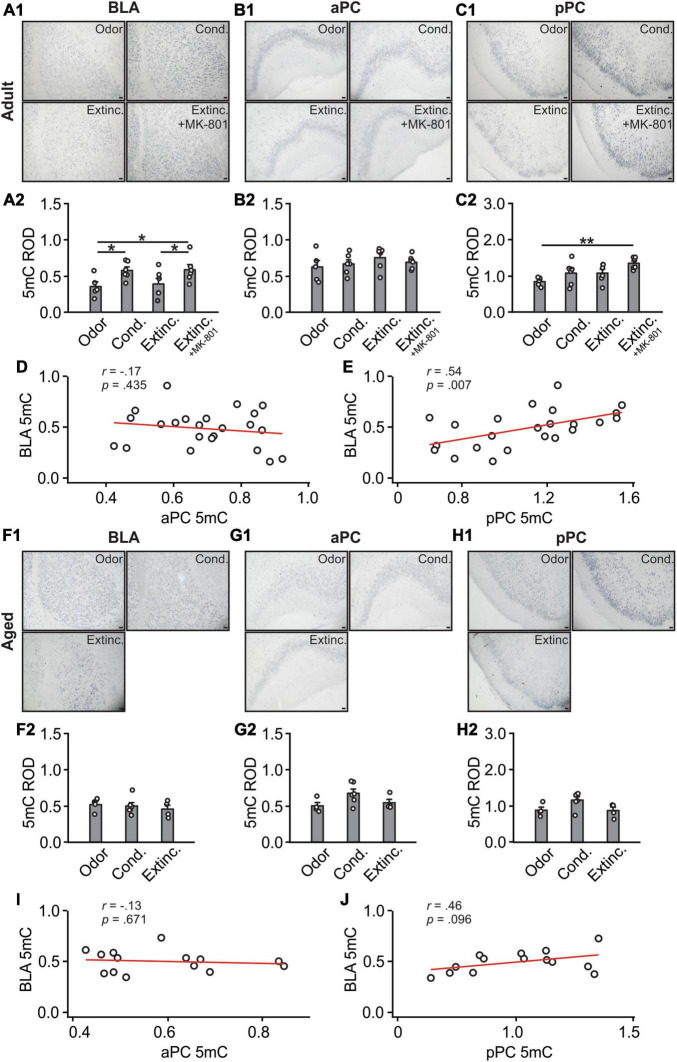
A 5mC activations in the BLA and pPC were strongly correlated in the adult rats. **(A1)** Example images of 5mC expression in the BLA of adult rats. **(A2)** 5mC relative optical density (ROD) in the BLA. **(B1)** Example images of 5mC expression in the aPC of adult rats. **(B2)** 5mC ROD in the aPC. **(C1)** Example images of 5mC expression in the pPC of adult rats. **(C2)** 5mC ROD in the pPC. **(D)** The correlation between BLA and aPC 5mC in adult rats. **(E)** The correlation between BLA and pPC 5mC in adult rats. **(F1)** Example images of 5mC expression in the BLA of aged rats. **(F2)** 5mC ROD in the BLA. **(G1)** Example images of 5mC expression in the aPC of aged rats. **(G2)** 5mC ROD in the aPC. **(H1)** Example images of 5mC expression in the pPC of aged rats. **(H2)** 5mC ROD in the pPC. **(I)** The correlation between BLA and aPC 5mC in aged rats. **(J)** The correlation between BLA and pPC 5mC in aged rats. Scale bars, 50 μm. *p < 0.05; **p < 0.01.

There were significant differences in 5mC expression in the BLA among adult groups (*F*(3,19) = 3.24, *p* = 0.045; Figures 4A1, A2), with the conditioned group showing significantly higher 5mC expression compared to odor only controls. The MK-801 injected group maintained a high level of 5mC and was significantly higher than the extinction-trained group without MK-801. Expression of 5mC were not different among groups in the aPC [*F*(3,19) = 0.69, *p* = 0.568; Figures 4B1, B2]. In the pPC, however, there were significant differences of 5mC among groups [*F*(3,19) = 4.34, *p* = 0.017; Figures 4C1, 4C2]. The MK-801 group had higher expression than the control odor only group. There was no correlation between aPC and BLA 5mC expression levels (*r* = −0.17, *p* = 0.435; Figure 4D), but a significant correlation was found between pPC and BLA (*r* = 0.54, *p* = 0.007; Figure 4E).

In aged rats, 5mC patterns in the BLA [*F*(3,19) = 0.33, *p* = 0.73; Figures 4F1, F2], aPC [*F*(3,11) = 2.838, *p* = 0.10; Figures 4G1, G2] and pPC [*F*(3,11) = 3.40, *p* = 0.07; Figures 4H1, H2] were not different among groups. There was neither correlation between aPC and BLA 5mC expression levels (*r* = −0.13, *p* = 0.671; Figure 4I), nor significant correlation between pPC and BLA (*r* = 0.46, *p* = 0.096; Figure 4J).

### 3.3 Histone H3K9/K14 acetylations were correlated between the BLA and pPC

**FIGURE 5:**
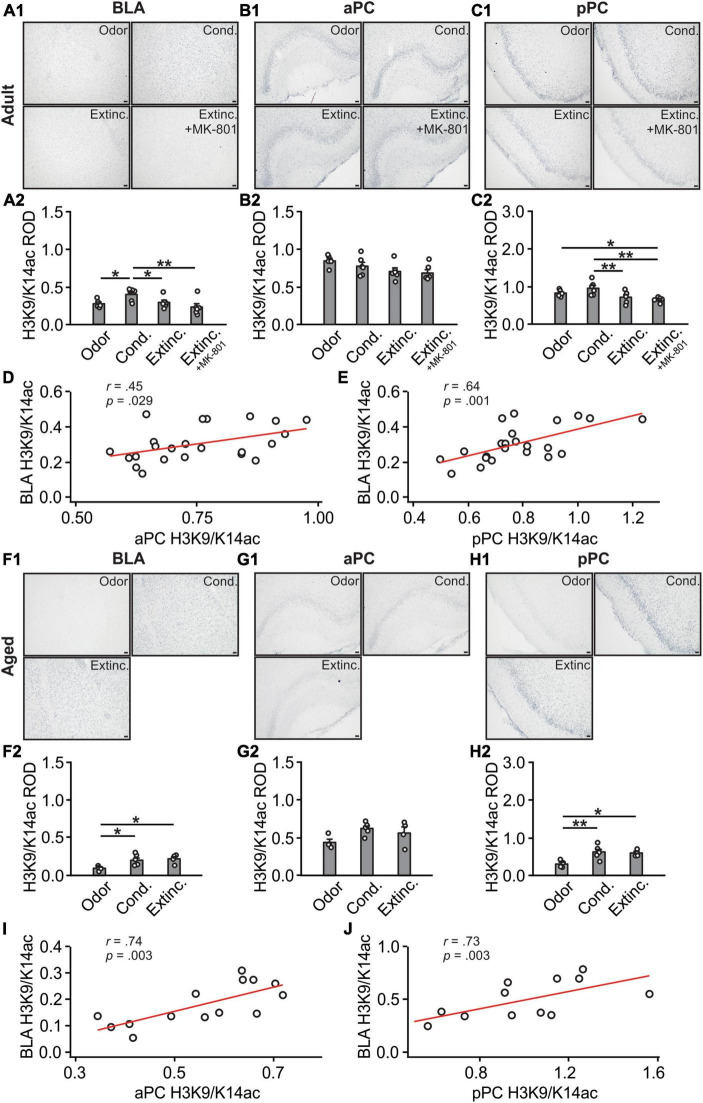
H3K9/K18 activations in the BLA and PC were strongly correlated. **(A1)** Example images of H3K9/K18ac expression in the BLA of adult rats. **(A2)** H3K9/K18ac ROD in the BLA. **(B1)** Example images of H3K9/K18ac expression in the aPC of adult rats. **(B2)** H3K9/K18ac ROD in the aPC. **(C1)** Example images of H3K9/K18ac expression in the pPC of adult rats. **(C2)** H3K9/K18ac ROD in the pPC. **(D)** The correlation between BLA and aPC H3K9/K18ac in adult rats. **(E)** The correlation between BLA and pPC H3K9/K18ac in adult rats. **(F1)** Example images of H3K9/K18ac expression in the BLA of aged rats. **(F2)** H3K9/K18ac ROD in the BLA. **(G1)** Example images of H3K9/K18ac expression in the aPC of aged rats. **(G2)** H3K9/K18ac ROD in the aPC. **(H1)** Example images of H3K9/K18ac expression in the pPC of aged rats. **(H2)** H3K9/K18ac ROD in the pPC. **(I)** The correlation between BLA and aPC H3K9/K18ac in aged rats. **(J)** The correlation between BLA and pPC H3K9/K18ac in aged rats. Scale bars, 50 μm. *p < 0.05; **p < 0.01.

H3K9/K14ac expression in the BLA were different among adult groups [*F*(3,19) = 4.29, *p* = 0.018; Figures 5A1, A2], with the conditioned group showing significantly higher H4K9/K14ac expression compared to the odor only and extinction groups. Interestingly, the extinction-trained group with MK-801 injection showed lower levels of H3K9/K14ac expression compared to the conditioned group. Expression of H3K9/K14ac were not significantly different among groups in the aPC (*F*(3,19) = 2.47, *p* = 0.093; Figures 5B1, B2). In contrast, in the pPC, there were significant differences of H3K9/K14ac among groups [*F*(3,19) = 5.86, *p* = 0.005; Figures 5C1, C2]. The extinction group had a significantly lower level of H3K9/K14ac compared to the conditioned group. However, similar to the BLA, the MK-801 injected group showed a low level of H3K9/K14ac in the pPC following the extinction training. Interestingly, there was a significant correlation between aPC and BLA H3K9/K14ac expression levels (*r* = 0.45, *p* = 0.029; Figure 5D), as well as a significant correlation between pPC and BLA (*r* = 0.64, *p* = 0.001; Figure 5E).

In aged rats, H3K9/K14ac patterns in the BLA were significantly different among groups [*F*(3,11) = 5.09, *p* = 0.027; Figures 5F1, F2] and were similar to the cFos patterns, with conditioned and extinction-trained groups showed higher levels of H3K9/K14ac expression. Expression levels in the aPC were not different among groups [*F*(3,11) = 3.54, *p* = 0.065; Figures 5G1, G2]. The pPC H3K9/K14ac patterns were similar to those in the BLA, with both the conditioned and extinction groups showing higher levels of expression [*F*(3,11) = 7.92, *p* = 0.007; Figures 5H1, H2]. Interestingly, there were significant correlations between aPC and BLA (*r* = 0.74, *p* = 0.003; Figure 5I), and between pPC and BLA H3K9/K14ac expression levels (*r* = 0.73, *p* = 0.003; Figure 5J).

### 3.4 Histone H3K27 acetylations were correlated between the BLA and pPC

**FIGURE 6:**
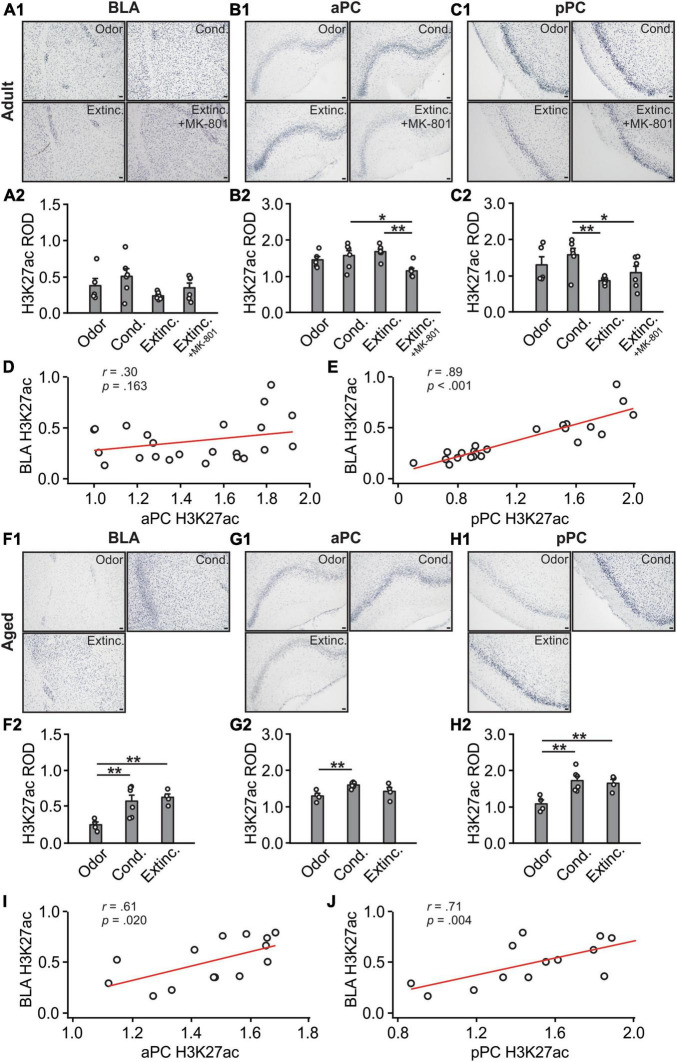
H3K27 activations in the BLA and pPC were strongly correlated. **(A1)** Example images of H3K27ac expression in the BLA of adult rats. **(A2)** H3K27ac ROD in the BLA. **(B1)** Example images of H3K27ac expression in the aPC of adult rats. **(B2)** H3K27ac ROD in the aPC. **(C1)** Example images of H3K27ac expression in the pPC of adult rats. **(C2)** H3K27ac ROD in the pPC. **(D)** The correlation between BLA and aPC H3K27ac in adult rats. **(E)** The correlation between BLA and pPC H3K27ac in adult rats. **(F1)** Example images of H3K27ac expression in the BLA of aged rats. **(F2)** H3K27ac ROD in the BLA. **(G1)** Example images of H3K27ac expression in the aPC of aged rats. **(G2)** H3K27ac ROD in the aPC. **(H1)** Example images of H3K27ac expression in the pPC of aged rats. **(H2)** H3K27ac ROD in the pPC. **(I)** The correlation between BLA and aPC H3K27ac in aged rats. **(J)** The correlation between BLA and pPC H3K27ac in aged rats. Scale bars, 50 μm. *p < 0.05; **p < 0.01.

H3K27ac expression in the BLA [*F*(3,19) = 1.99, *p* = 0.149; Figures 6A1, A2] were not different among adult groups. In the aPC [*F*(3,19) = 5.12, *p* = 0.009; Figures 6B1, 6B2], the H3K27ac expression in the MK-801 group were significantly lower than those in the conditioned and extinction-trained groups. In the pPC, there were significant differences in H3K27ac among groups [*F*(3,19) = 3.53, *p* = 0.035; Figures 6C1, C2]. Both the extinction and the MK-801 groups had significantly lower levels of H3K27ac compared to the conditioned group. There was no correlation between aPC and BLA H3K27ac expression levels (*r* = 0.30, *p* = 0.166; Figure 6D), but significant correlation was observed between pPC and BLA H3K27ac levels (*r* = 0.89, *p* < 0.001; Figure 6E).

In aged rats, significant differences of H3K27ac expression were observed in all the structures, BLA [*F*(2,11) = 7.44, *p* = 0.009; Figures 6F1, F2], aPC [*F*(2,11) = 5.40, *p* = 0.023; Figures 6G1, G2] and pPC [*F*(2,11) = 8.63, *p* = 0.006; Figures 6H1, H2]. The H3K27ac expression were significantly correlated between the aPC and BLA (*r* = 0.61, *p* = 0.020; Figure 6I), and between the pPC and the BLA (*r* = 0.71, *p* = 0.004; Figure 6J).

### 3.5 Histone H4 acetylations were correlated between the BLA and pPC in adult rats only

**FIGURE 7:**
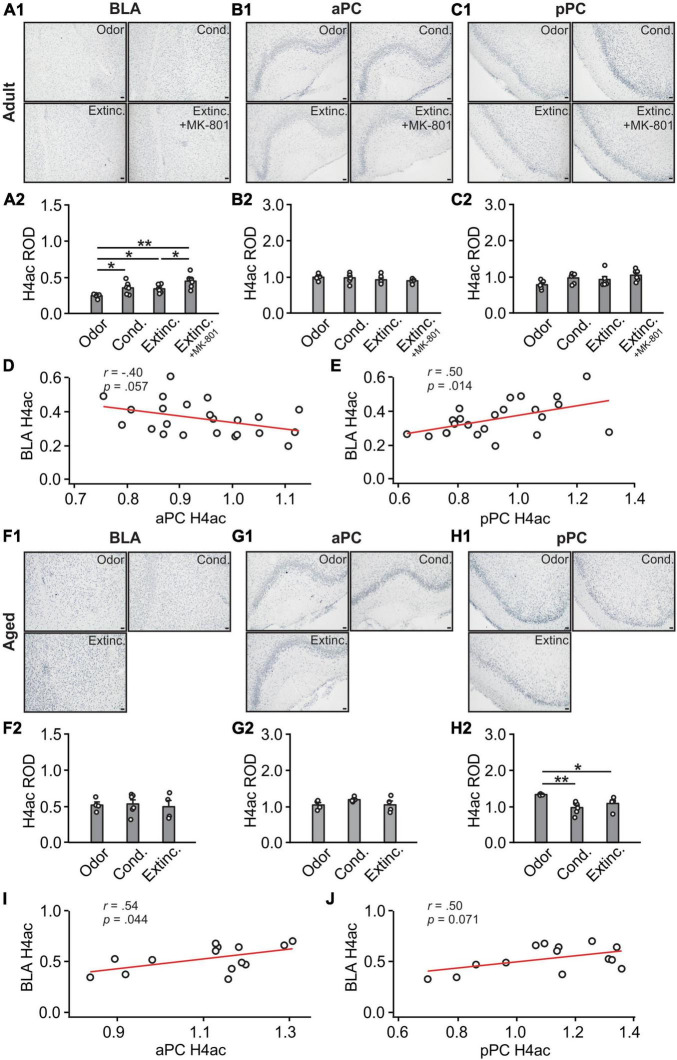
H4 activations in the BLA and pPC were strongly correlated in adult rats. **(A1)** Example images of H4ac expression in the BLA of adult rats. **(A2)** H4ac ROD in the BLA. **(B1)** Example images of H4ac expression in the aPC of adult rats. **(B2)** H4ac ROD in the aPC. **(C1)** Example images of H4ac expression in the pPC of adult rats. **(C2)** H4ac ROD in the pPC. **(D)** The correlation between BLA and aPC H4ac in adult rats. **(E)** The correlation between BLA and pPC H4ac in adult rats. **(F1)** Example images of H4ac expression in the BLA of aged rats. **(F2)** H4ac ROD in the BLA. **(G1)** Example images of H4ac expression in the aPC of aged rats. **(G2)** H4ac ROD in the aPC. **(H1)** Example images of H4ac expression in the pPC of aged rats. **(H2)** H4ac ROD in the pPC. **(I)** The correlation between BLA and aPC H4ac in aged rats. **(J)** The correlation between BLA and pPC H4ac in aged rats. Scale bars, 50 μm. *p < 0.05; **p < 0.01.

H4ac expression in the BLA were different among groups [*F*(3,19) = 6.74, *p* = 0.003; Figures 7A1, A2]. Conditioned and MK-801 extinction groups had higher levels of H4Ac than the odor only and extinction groups. H4ac expression in the aPC [*F*(3,19) = 1.09, *p* = 0.377; Figures 7B1, B2] and pPC [*F*(3,19) = 2.62, *p* = 0.080; Figures 7C1, C2] were not different among adult groups. There was no correlation between aPC and BLA H3K27ac expression levels (*r* = −0.40, *p* = 0.057; Figure 7D), however, significant correlation was found between pPC and BLA H4ac (*r* = 0.50, *p* = 0.014; Figure 7E).

In aged rats, there were no trends of differences in H4ac expression in the BLA (*F*(2,11) = 0.09, *p* = 0.911; Figures 7F1, 6F2) and aPC (*F*(2,11) = 1.995, *p* = 0.182; Figures 7G1, G2). However, oddly, there was reduced expression of H4ac in the conditioned group in the pPC (*F*(2,11) = 6.68, *p* = 0.013; Figures 7H1, H2). H4ac expression was correlated between the aPC and BLA (*r* = 0.54, *p* = 0.044; Figure 7I) and less correlated between the pPC and BLA (*r* = 0.50, *p* = 0.071; Figure 7J).

### 3.6 Correlations between epigenetic markers and cFos expression in the BLA and pPC

We further analyzed correlations of different epigenetic markers with cFos in the pPC and BLA (Table 1). In adult rats, the MK-801 group included, there was a significant correlation between 5mC and cFos in the BLA (*r* = 0.44, *p* = 0.036). Excluding MK-801, a significant correlation between BLA 5mC and cFos remained (*r* = 0.51, *p* = 0.038). Additionally, the H3K9/K14ac was strongly correlated with cFos in the BLA (*r* = 0.52, *p* = 0.031). There were no strong correlations with cFos in BLA with expression of H3K27ac or H4ac. There were also no significant correlations in all pPC epigenetic markers with cFos in adult rats.

**TABLE 1:** The correlations between epigenetic markers and cFos in the BLA and pPC.

Correlation with cFos	Adult		Adult (excl. MK-801)		Aged	
	**pPC**	**BLA**	**pPC**	**BLA**	**pPC**	**BLA**
5mC	*r* = 0.35	*r* = 0.44	*r* = 0.23	*r* = 0.51	*r* = 0.27	*r* = 0.004
	*p* = 0.099	*p = 0.036	*p* = 0.382	*p = 0.038	*p* = 0.345	*p* = 0.999
H3K9/K14ac	*r* = -0.02	*r* = 0.15	*r* = 0.24	*r* = 0.52	*r* = 0.68	*r* = 0.38
	*p* = 0.876	*p* = 0.504	*p* = 0.353	*p = 0.031	**p = 0.007	*p* = 0.184
H3K27ac	*r* = 0.19	*r* = 0.33	*r* = 0.28	*r* = 0.44	*r* = 0.67	*r* = 0.55
	*p* = 0.376	*p* = 0.120	*p* = 0.28	*p* = 0.075	**p = 0.008	*p = 0.041
H4ac	*r* = 0.29	*r* = 0.60	*r* = 0.25	*r* = 0.40	*r* = -0.35	*r* = 0.20
	*p* = 0.183	*p* = 0.097	*p* = 0.33	*p* = 0.112	*p* = 0.214	*p* = 0.500

*p < 0.05;

**p < 0.01.

In aged rats, differential patterns of correlations emerged. H3K27ac in the BLA had significant correlation with cFos (*r* = 0.55, *p* = 0.041), whereas H3K9/K14ac (*r* = 0.68, *p* = 0.007) and H3K27ac (*r* = 0.67, *p* = 0.008) in the pPC showed significant correlations with cFos (Table 1).

### 3.7 Age-dependent changes in epigenetic activation

**FIGURE 8:**
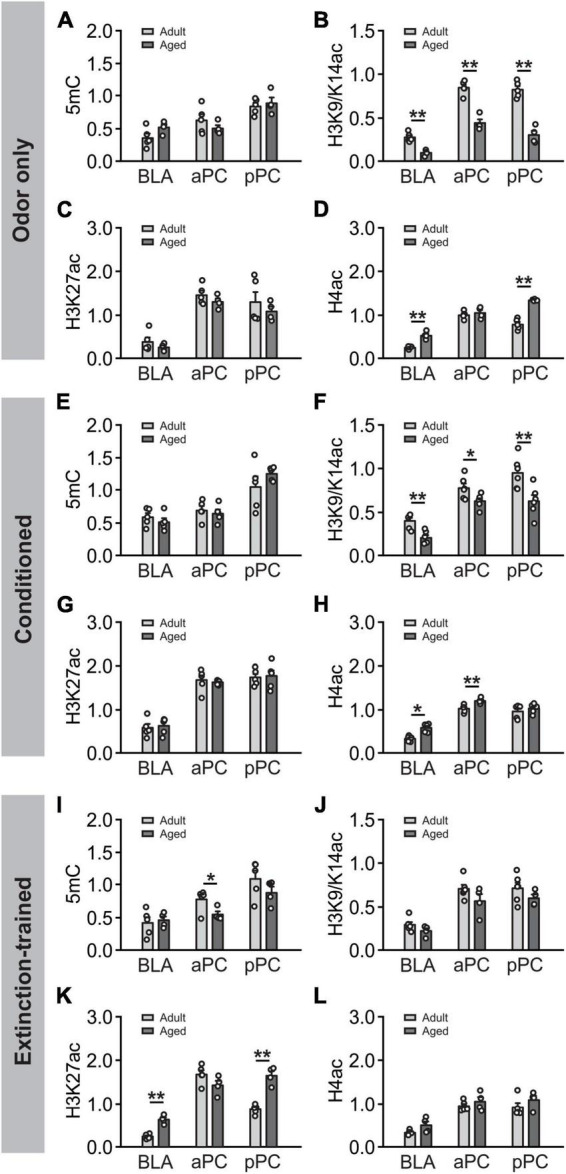
Age-dependent DNA methylation and histone acetylation. **(A–D)** Comparisons of age differences in epigenetic expression in the odor only condition. Panel **(A)** Comparison of 5mC expression in the BLA, aPC and pPC in two age groups. Panel **(B)** Comparison of H3K9/K14ac expression in two age groups. Panel **(C)** Comparison of H3K27ac expression in two age groups. Panel **(D)** Comparison of H4ac expression two age groups. **(E–H)** Comparisons of age differences in epigenetic expression following olfactory conditioning: 5mC Panel **(E)**, H3K9/K14ac Panel **(F)**, H3K27ac Panel **(J)** and H4ac Panel **(H)**. **(I–L)** Comparisons of age differences in epigenetic expression following extinction training: 5mC **(I)**, H3K9/K14ac **(J)**, H3K27ac **(K)** and H4ac **(L)**. *p < 0.05; **p < 0.01.

We next compared the epigenetic expression in adult and aged rats (Figure 8). At basal odor only condition (Figures 8A–D), no differences were found in 5mC expression in the two age groups at all areas (Figure 8A). However, significantly lower levels in H3K9/K14ac were observed in the aged group in all structures, BLA (*t* = 5.79, *p* < 0.001), aPC (*t* = 7.41, *p* < 0.001) and pPC (*t* = 8.26, *p* < 0.001) (Figure 8B). H3K27ac expression were similar in the two age groups in all areas (Figure 8C). Noteworthy, H4ac levels were increased in the aged group in the BLA (*t* = 6.71, *p* < 0.001), pPC (*t* = 8.96, *p* < 0.001) but not aPC (*t* = 0.73, *p* = 0.490) (Figure 8D). Following olfactory threat conditioning (Figures 8E–H), the patterns of age differences were similar to those without learning. No age differences were observed for 5mC (Figure 8E). Lower levels of H3K9/K14ac were observed in the aged group in all areas, BLA (*t* = 4.316, *p* = 0.002), aPC (*t* = 2.503, *p* < 0.031) and pPC (*t* = 3.222, *p* = 0.009; Figure 8F). No age differences were observed in H3K27ac expression (Figure 8G). Higher expression of H4ac were observed in the BLA (*t* = 2.774, *p* = 0.020) and aPC (*t* = 3.684, *p* = 0.004; Figure 8H). Intriguingly, following extinction training, different patterns emerged (Figures 8I–L). 5mC expression were not different in the two age groups in the BLA and pPC, but were in the aPC (*t* = 2.347, *p* = 0.047; Figure 8I). H3K9/K14ac expression were not different in the two age groups in any of the structures (Figure 8J). However, H3K27ac expression showed differences between the two ages (Figure 8K), with higher levels observed in the BLA (*t* = 8.484, *p* < 0.001) and pPC (*t* = 8.290, *p* < 0.001). H4ac expression were not different in the two age groups (Figure 8L). As aged rats did not distinguish the olfactory threat memory, the difference in epigenetic expression following extinction learning may reflect a difference related to behavioral outcomes rather than age.

## 4 Discussion

In this study, we systemically surveyed cFos and epigenetic expression in the BLA and PC following olfactory threat conditioning and extinction learning. Firstly, we found that patterns of cFos expression in both the BLA and pPC mirrored the freezing behavior observed by the subjects. In adult rats, cFos expression in the BLA and pPC was elevated following odor threat conditioning, but returned to baseline levels after extinction training. Administration of the NMDAR antagonist MK-801 prevented extinction learning and preserved high levels of cFos^+^ nuclei in both structures. In aged rats, extinction training failed to extinguish the odor threat memory. In parallel, cFos expression in the pPC maintained high levels following the extinction training, comparable to those following the olfactory threat conditioning. Furthermore, cFos expression levels were highly correlated between the BLA and pPC in both age groups. In contrast, aPC cFos patterns correlated with neither behavioral freezing nor BLA cFos patterns.

The BLA has been posited as the central element of threat learning and extinction. Interestingly, increased activation in response to conditioned cues has been observed following both threat conditioning and extinction learning in human and rodent amygdala (LaBar et al., 1998; Sehlmeyer et al., 2009; Siddiqui et al., 2017), but see (Zeidan et al., 2011). However, the activation of the amygdala appears to be transient and is not observed in the late phases of fear acquisition and extinction in humans (LaBar et al., 1998). Our cFos measurement was conducted following 6 days of extinction training and likely correlates with the late phase of human fear extinction. The differences in cFos activation following extinction learning in animal models could be accounted for by different training paradigms, and phases of the training, which are associated with the balance between excitation and inhibition of several neuronal populations. Distinct subsets of neurons in the amygdala exist for threat acquisition and extinction (Herry et al., 2008). Additionally, extinction could be mediated by inhibitory neurons within the amygdala (Likhtik et al., 2008) which suppress the activity of excitatory principal neurons. Alternately, reduced cFos expression following successful extinction learning in our study could reflect depotentiation, or LTD, of excitatory synapses of the principal neurons (Lin et al., 2003; Kim et al., 2007; Sepahvand et al., 2023a).

One novel finding in this study is that BLA and pPC neuronal activities indexed by cFos, are highly synchronized in odor threat conditioning and extinction learning. Similar to hippocampus-dependent memory that undergoes substantial system consolidation, which engages cortical areas for remote memory storage (Frankland and Bontempi, 2005), recent evidence has portrayed BLA as the initial encoding structure for emotional learning, while sensory cortices serve as long-term storage of emotional memory (Sacco and Sacchetti, 2010; Cambiaghi et al., 2016a; Mouly et al., 2022; You et al., 2022; Li and Wilson, 2023). This theory is supported anatomically by the existence of strong mutual connections between the pPC and BLA (Majak et al., 2004). As such, network oscillations between the BLA and the pPC appear to promote information transfer during the formation of olfactory threat memory (East et al., 2021). Recently, we have reported that pPC is a locus for olfactory threat extinction (Sepahvand et al., 2023a). Successful extinction of olfactory threat memory is associated with NMDAR-dependent LTD in the pPC (Sepahvand et al., 2023a). In this work, we further demonstrated the synchrony of neuronal activity between the BLA and pPC, during both olfactory threat acquisition and extinction. Our results further support that the associative sensory cortices are the final repertoire of emotional memory storage.

**FIGURE 9:**
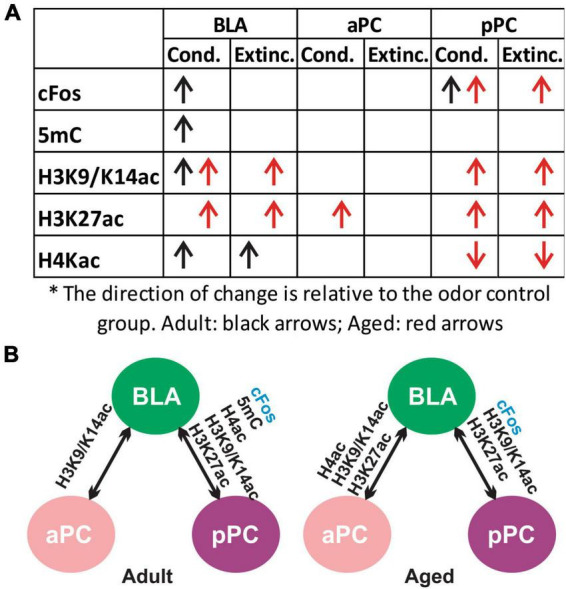
Summary of cFos and epigenetic expression in olfactory threat conditioning and extinction learning. **(A)** Changes of cFos and epigenetic markers in the conditioned and extinction-trained groups compared to the odor only group (*post hoc* Tukey followed by one-way ANOVA). **(B)** cFos and epigenetic mark correlations between the BLA, aPC and pPC in adult and aged rats.

Secondly, we found that epigenetic activity was largely correlated between the BLA and pPC, even though each marker exhibited distinct, age-dependent patterns of activation following olfactory threat conditioning and extinction learning (Figure 9A). This was evident in adult rats, as there were strong correlations between the BLA and pPC in all the markers tested (5mC, H3K9K14ac, H3K27ac and H4ac; Figure 9B). In aged rats, strong correlations were observed in H3 acetylation between the BLA and pPC. Interestingly, with aging, aPC epigenetic expression appeared to correlate more strongly with BLA versus being in the youthful state. H3 and H4 acetylation in the aPC were strongly correlated with those in the BLA in the aged group, but not in the adult group. Hyperexcitability of the BLA in aging subjects (Prager et al., 2016) may shift the interaction and dynamic of neural modulation in the PC.

Paramount data has suggested that chromatin modifications such as those induced by DNA methylation and histone acetylation are a crucial mechanism for threat memory acquisition, retrieval and extinction (Miller and Sweatt, 2007; Graff and Tsai, 2013; Zovkic et al., 2013; Guzman-Karlsson et al., 2014; Ell et al., 2023). Animal studies demonstrate both global and gene specific modifications of DNA methylation and histone acetylation in threat learning (Bredy et al., 2007; Monsey et al., 2011; Itzhak et al., 2012; Halder et al., 2016; Siddiqui et al., 2017). Intriguingly, these modifications have been shown to be a dynamic function, dependent on the time, phase of training, and brain regions involved (Levenson et al., 2004; Gupta et al., 2010; Ell et al., 2023). In the BLA, auditory threat conditioning is associated with increased H3 and H4 acetylation and DNA methylation enzyme DNMT3a (Monsey et al., 2011; Siddiqui et al., 2017). Elevated H3 and H4 acetylation levels in the BLA by histone deacetylase inhibitors are associated with enhanced contextual threat learning, and extinction of auditory threat memory, respectively (Itzhak et al., 2012). In Siddiqui et al. (2017) study, elevated H3K9 acetylation was observed following both auditory threat conditioning and extinction in the rat BLA, paralleled by enhanced cFos expression in both conditions. Here we show in adult rats that H3K9/K14 expression were elevated following threat conditioning but were diminished by extinction training. Furthermore, in aged rats, H3 acetylation was maintained at high levels following failed extinction learning. These results suggest that in our olfactory threat paradigm, successful extinction is associated with reduced H3 acetylation in the BLA, in contrast to the study by Siddiqui et al. (2017). Consistent with our study, in a mouse model of contextual threat extinction (Bahari-Javan et al., 2012), facilitated extinction by HDAC1 overexpression is associated with reduced cFos and H3K9 acetylation in the hippocampus. The differences in the modality (olfactory versus auditory) and training protocols (multi-day training versus one day) could contribute to the differential results observed between Siddiqui et al. (2017) study and ours. Indeed, both multi-day contextual extinction in mice (Bahari-Javan et al., 2012) and multi-day olfactory extinction training in our rat study yielded the same results in terms of reduced cFos and H3K9ac activation. Similar to the cFos profiles following learning (LaBar et al., 1998), histone modification also displays a transient nature and only occurs within a few hours following memory consolidation (Levenson et al., 2004; Gupta et al., 2010). Nevertheless, the changes in H3 acetylation in our study, including H3K9/K14 and H3K27, were generally correlated with the degree of cFos expression in the BLA and pPC in relation to olfactory threat acquisition and extinction. Levels of H4 acetylation in the BLA also paralleled cFos in adult rats but did not reach a significant correlation. H4ac expression was highly correlated between the BLA and pPC in adult rats, but not in the aged subjects.

5mC expression in the BLA correlated with cFos patterns in adult rats, but not in aged rats. The correlation between 5mC and cFos was weaker in the pPC at both ages. Additionally, 5mC expression were highly correlated between the BLA and pPC in adult rats, while the correlations between the two structures were weaker in aged rats. DNA methylation and demethylation are critically involved in threat memory acquisition and extinction. Hypermethylation of plasticity-related molecules in the ten-eleven translocation (Tet) knockout mice, is associated with impaired hippocampus-dependent contextual and spatial memory extinction, despite that learning acquisition remains normal in these mice (Rudenko et al., 2013). Conversely, overexpression of Tet-1 in the dorsal hippocampus is associated with impaired contextual memory expression (Kaas et al., 2013). Tet-1 is an enzyme that converts 5mC to 5-hydroxymethlcytosin (5hmC) (Tahiliani et al., 2009), thus it promotes DNA demethylation. Enhanced 5mC expression following olfactory threat conditioning, and reduced DNA methylation following successful extinction learning in adult rats in our study, are consistent with the requirement of proper levels of DNA methylation in supporting memory acquisition and extinction (Kaas et al., 2013; Rudenko et al., 2013). Keeping consistent with our study, a lower level of amygdala 5hmC (suggesting hypermethylation) in another rat model is also associated with impaired threat extinction. In our aged rats, failed extinction learning is also correlated with a sustained high level of DNA methylation 5mC.

Intriguingly, extinction training with MK-801 injections enhanced 5mC and H4ac levels in adult rats, paralleling behavioral freezing scores and cFos changes, whereas H3 acetylation showed reduced activation following the same training with MK-801. NMDAR blockade may differentially influence various chromatin modifications associated with memory reconsolidation and extinction, or other aspects not directly related to learning.

Finally, we compared epigenetic expression at two ages. At basal odor only condition without learning, we observed decreased expression of H3K9/K14ac in all areas and increased expression of H4ac in the BLA and pPC in the aged rats. The age-dependent differences in epigenetic patterns were similar following the olfactory threat conditioning, with lower levels of H3K9/K14ac and higher levels of H4ac. Intriguingly, epigenetic expression patterns were distinct following the threat extinction training at the two ages. Higher levels of H3K27ac were observed in the BLA and pPC in the aged animals. Sustained high level of H3K27ac in aged animals may relate to the failure of extinction learning.

Numerous studies have reported that alterations in DNA methylation and histone acetylation are associated with aging and aging-related cognitive decline (Maity et al., 2021). Comparing an aging-accelerated mouse line, senescence accelerated mouse-prone 8 (SAMP2), with an aging resistant mouse line, Cosin-Tomas et al. (Cosín-Tomás et al., 2018) shows global decrease in DNA methylation and an increase in H3 and H4 acetylation in the hippocampus associated with aging. Our results suggest brain region-specific alteration of chromatin modifications occurs with aging. Gene-specific modifications of DNA methylation and histone acetylation, however, exhibit distinct patterns with aging (Maity et al., 2021). For instance, 5mC has been shown to be increased at genes related to synaptic plasticity in the prefrontal cortex (Ianov et al., 2017) and hippocampus (Penner et al., 2011, 2016) in aged rodents, which corresponds to the regression of these genes and associated cognitive decline. Global DNA hypomethylation promotes entropy in aging (Kane and Sinclair, 2019) while plasticity gene-specific increase in DNA methylation may decrease “signal to noise” ratio for learning-associated molecules. On the other hand, histone downregulation has been associated with aging and aging-associated memory decline. For instance, H3K12 acetylation is down-regulated in the hippocampus in mice and is associated with HDAC hyperfunction (Peleg et al., 2010). Inhibition of HDACs restores histone acetylation in the brain and rescues learning impairment in aged animals (Peleg et al., 2010; Singh and Thakur, 2018). Remarkably, our results demonstrate region-specific, differential regulations of H3 and H4 acetylation with no difference in 5mC. It remains pertinent to further explore how histone acetylation and DNA methylation regulate specific genes salient for learning and plasticity throughout the aging process.

In summary, our study highlights the complex and dynamic nature of epigenetic modifications during learning and aging. DNA methylation and histone modifications may work in a concerted fashion to regulate the transcription of genes that are crucial for memory formation and extinction. The cross-talk demonstrated between epigenetic marks makes them reciprocally regulating (Gupta et al., 2010; Parrish et al., 2010; Ell et al., 2023) which is a complexity that potentially occurs during any type of learning. The strong correlation seen between epigenetic marks in the BLA and pPC in adult rats during associative learning and extinction, and their correlations with neuronal activity indexed by cFos, support the view that both BLA and PC are critical memory encoding and storage structures (Li and Wilson, 2023). A larger interaction, which includes the correlated epigenetic changes between the aPC and BLA of aged brains, may implicate a shift to a less specific epigenetic dynamic emerging with aging.

## Data availability statement

The original contributions presented in this study are included in this article/Supplementary material, further inquiries can be directed to the corresponding author.

## Ethics statement

The animal study was approved by the Institutional Animal Care Committee at Memorial University of Newfoundland. The study was conducted in accordance with the local legislation and institutional requirements.

## Author contributions

TQ: Data curation, Formal Analysis, Methodology, Validation, Writing−original draft, Writing−review and editing. YX: Data curation, Formal Analysis, Methodology, Validation, Writing−original draft, Writing−review and editing. NN: Data curation, Methodology, Writing−review and editing. TS: Data curation, Methodology, Writing−review and editing. QY: Conceptualization, Data curation, Formal Analysis, Funding acquisition, Methodology, Project administration, Resources, Supervision, Validation, Visualization, Writing−original draft, Writing−review and editing.
