# Supplementary material for: Correlational patterns of neuronal activation and epigenetic marks in the basolateral amygdala and piriform cortex following olfactory threat conditioning and extinction in rats
Source: Front Mol Neurosci. 2024 Mar 14;17:1355140. doi: 10.3389/fnmol.2024.1355140 (PMC10972882; doi:10.3389/fnmol.2024.1355140)
Supplement: Supplementary file 1 [file Data_Sheet_1.PDF]

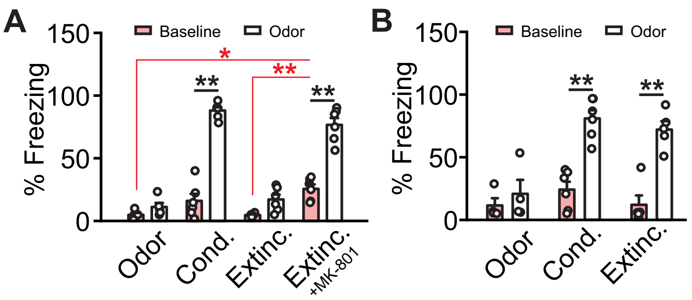

Supplementary Figure 1. Comparison of baseline freezing (5 min) and freezing in the presence of the odor. **A.** Comparison of the percent freezing in various groups in adult rats. **B.** Comparison of the percent freezing in various groups in aged rats. Two-way repeated ANOVAs were used to compare the Group X Time (baseline vs. odor) interaction. Post-hoc Tukey tests were used to compare between groups. *$p < 0.05$, **$p < 0.01$.
